# HIV/AIDS and Associated Conditions among HIV-Infected Refugees in Minnesota, 2000–2007 

**DOI:** 10.3390/ijerph9114197

**Published:** 2012-11-16

**Authors:** Sara A. Lowther, Glenise Johnson, Brett Hendel-Paterson, Kailey Nelson, Blain Mamo, Kristina Krohn, Luisa Pessoa-Brandão, Ann O’Fallon, William Stauffer

**Affiliations:** 1 Centers for Disease Control and Prevention, 1600 Clifton Road, MS A-04, Atlanta, GA 30333, USA; 2 Minnesota Department of Health (MDH), Saint Paul, MN 55164, USA; Email: glenise.johnson@state.mn.us (G.J.); kailey.nelson@state.mn.us (K.N.); blain.mamo@state.mn.us (B.M.); lpessoa-brandao@nastad.org (L.P.-B.); ann.ofallon@gmail.com (A.O.F.); 3 Department of Medicine—Global Health, University of Minnesota, Minneapolis, MN 55455, USA; Email: bhendel1@gmail.com (B.H.-P.); kmkrohn@gmail.com (K.K.); stauf005@umn.edu (W.S.); 4 HealthPartners Regions Hospital, Saint Paul, MN 55104, USA; 5 Division of Global Migration and Quarantine, Centers for Disease Control and Prevention, Atlanta, GA 30333, USA

**Keywords:** HIV, acquired immunodeficiency syndrome, refugees, emigration and immigration, epidemiology

## Abstract

In 2010, the requirement for human immunodeficiency virus (HIV) testing of adult refugees prior to US resettlement was removed, thus leading to a potential for missed diagnosis. We reviewed refugee health assessment data and medical charts to evaluate the health status of HIV-infected refugees who arrived in Minnesota during 2000–2007, prior to this 2010 policy change. Among 19,292 resettled adults, 174 were HIV-infected; 169 (97%) were African (median age 26.4 (range: 17–76) years). Charts were abstracted for 157 (124 (79%) with ≥1 year of follow-up). At initial presentation, two of 74 (3%) women were pregnant; 27% became pregnant during follow-up. HIV clinical stage varied (59%, asymptomatic; 11%, mild symptoms; 10%, advanced symptoms; 3%, severe symptoms; 17%, unknown); coinfections were common (51 tuberculosis, 13 hepatitis B, 13 parasites, four syphilis). Prior to arrival 4% had received antiretrovirals. Opportunistic infections were diagnosed among 13%; 2% died from AIDS-related causes. Arrival screening may be needed to identify these HIV-infected refugees and prevent HIV-related morbidity and mortality.

## 1. Introduction

The US Refugee Resettlement Program actively resettles 34,000–119,000 refugees annually [1,2]. Refugees face challenges to their physical and mental health. Before migration, refugees undergo medical screening for diseases of public health concern. Certain conditions are considered excludible by US law. These conditions, when identified, must either be treated successfully or the refugee must apply for a waiver that allows immigration despite the condition (US State Department Class A waiver). Upon arrival in the United States, all refugees are encouraged to have a domestic health screening assessment [3]. Medical screening includes obtaining a general medical history and physical examination, screening for infections (e.g., intestinal parasites and hepatitis B), and provision of preventive services (e.g., vaccinations) and counseling [3]. Each state is notified of newly arriving refugees and facilitates medical follow-up, with particular attention paid to refugees who arrive with Class A conditions. In Minnesota, surveillance data from the initial refugee medical screening are collected by the Minnesota Department of Health (MDH). These data are evaluated and used to guide public health prevention and control activities [4].

During 1993–2009, human immunodeficiency virus (HIV) infection was listed as a communicable disease of public health importance and an excludible condition for entry to the United States [5]. Refugees were allowed to migrate to the United States despite the HIV infection for humanitarian reasons after obtaining a Class A waiver. The Class A waiver notification system alerted states and health care providers to underlying medical conditions for refugees [5]. During 2000–2009, Minnesota was among states that participated in the resettlement waiver program for refugees with HIV infection. On 2 November 2009, the Department of Health and Human Services (DHHS) and Centers for Disease Control and Prevention (CDC) published a final rule that removed HIV infection from the list of communicable diseases of public health significance [5]. Beginning January 2010, HIV testing was removed from the overseas medical examination before arrival in the United States. CDC has issued guidance that highly encourages post-arrival medical screening and testing for HIV-infection among refugees [6].

Epidemiologic and clinical data regarding arriving HIV-infected refugees before the US government rule change have been limited [7,8,9]. As a supplemental surveillance project, epidemiologic and clinical data on arrival and during ≥1 year of follow-up care were collected for HIV-infected refugees who resettled to Minnesota during 2000–2007. We sought to describe these refugees’ stage of HIV infection, the burden of concomitant disease, and the initial clinical course. Because the impact of the rule change on the health of refugees arriving in the United States is unknown, these data can provide baseline information for subsequent evaluation of the impact of the rule change on refugee health status after US arrival.

## 2. Methods

### 2.1. Participants

Eligible participants included adults (aged ≥ 17 years) who arrived with known HIV infection or who received an HIV diagnosis after arrival. All refugees were primary arrivals, resettled to Minnesota from abroad without living elsewhere in the United States. Multiple methods were used to identify HIV-infected refugees to ensure inclusion of all eligible participants. Refugees were identified through the MDH refugee database screening for any refugee with Class A conditions reported through the US Department of State. Refugees identified with HIV infection during the post-arrival refugee screening were reported to MDH. Refugees who arrived in Minnesota but did not receive new arrival medical screening were identified through MDH HIV surveillance. 

### 2.2. Data Collection

In Minnesota, an initial refugee health assessment is recommended within 90 days of arrival. MDH recommends that providers review general health history, perform a physical examination, evaluate immunization status and provide vaccinations as needed, and perform screening for prevalent conditions encountered (e.g., tuberculosis (TB), hepatitis B, intestinal parasites by stool examination, lead poisoning for children under 6 years of age, malaria, dental, vision, hearing, and mental health). We included health data on new arrivals that were directly reported to the state of Minnesota. By using a data collection tool, additional data were abstracted from the follow-up HIV referral clinic that provided ongoing care to the refugee. We included demographic information, the stage of HIV infection, comorbidities, antiretroviral treatment before and after arrival, and clinical course after arrival. 

### 2.3. Measures

Medical chart abstractors categorized the patient into a World Health Organization (WHO) clinical stage [10] on the basis of their health condition documented in medical records at their first clinical visit after arrival (stage I, asymptomatic; stage II, mild disease; stage III, advanced disease; or stage IV, severe disease). Since 1991 and during the arrival of HIV-infected refugees described here, Class B1 TB was defined as an abnormal chest radiograph, indicating inactive TB with negative sputum smears or suspicion of extrapulmonary TB. In 2007, Class B1 TB was merged with Class B2 to include TB that is not clinically active and not infectious; old, healed TB and previously treated TB are also included. 

### 2.4. Analysis

The analyses are primarily descriptive. We examined demographic information and described co-morbidities such as the proportions with TB, hepatitis B infection, parasitic infections, and sexually transmitted infections noted on the domestic refugee health assessment. In addition, longitudinal data after arrival was examined for outcome data such as successful integration into ongoing care, and ongoing or new morbidity and mortality due to HIV infection. We also compared the types and prevalence of coinfections among newly arrived refugees who were HIV-infected versus HIV-uninfected arriving during the same period. For this comparison, if a refugee tested positive for HIV during the overseas exam but was re-tested and found to be negative in the States, they were categorized as HIV-uninfected; conversely, if a refugee tested negative for HIV overseas but was re-tested and found to be positive in the US, they were included as HIV-infected. 

We used *Χ*^2^-test or Fisher’s exact test when expected values were ≤ 5. Data were managed with Microsoft^®^ Access^®^ (Microsoft Corporation, Redmond, WA, USA). Statistical analyses were conducted by using SAS^®^ Version 9.1 (SAS Institute, Inc., Cary, NC, USA) and Stata^®^ 10 (StataCorp LP, College Station, TX, USA).

### 2.5. Ethics

Each participating institution that provided care or data considered this chart review and analysis to be a supplementary public health surveillance activity and determined it not to be human subjects research; therefore, approval by an institutional review board and informed consent were not required.

## 3. Results

**Figure 1:**
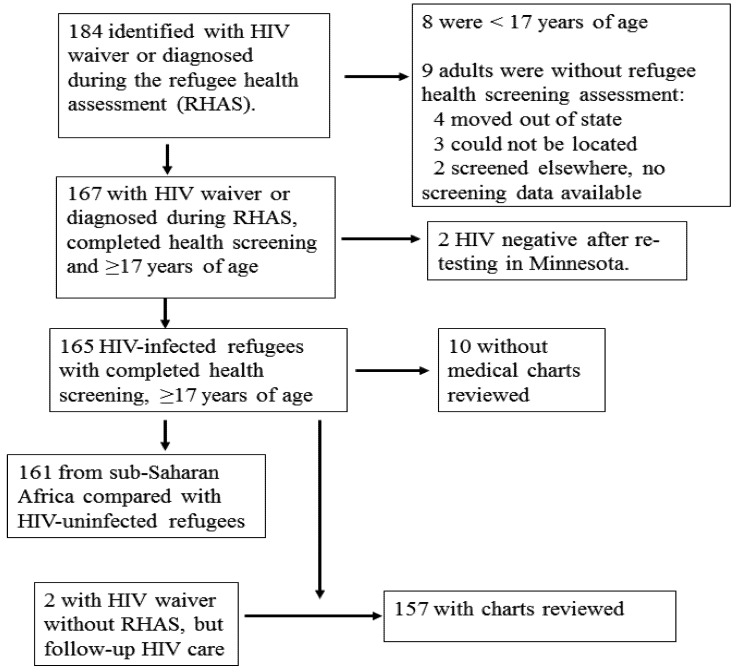
Refugees Entering Minnesota During 2000–2007 Who Had HIV Waivers or Were Diagnosed During Refugee Screening Identified as HIV-Infected Upon Further Testing and with Medical Charts Available for Review.

During 1 January 2000–31 December 2007, a total of 31,141 refugees were resettled to Minnesota, among whom 19,292 were adults aged ≥17 years, and 17,013 completed the initial refugee health assessment. There were 174 adult refugees identified as having HIV. One hundred fifty-seven (85%) had medical charts reviewed and were eligible for inclusion (Figure 1, Table 1). This included two adult refugees with an HIV waiver who missed their refugee health screening, but received follow-up HIV care.

Characteristics of the HIV-infected refugees identified through health screening and those with or without medical charts are described in Table 1. Among the 157 persons with medical charts reviewed, 83 (53%) indicated they were married or had a partner (49 had a partner with unknown HIV status). Among the 83 who had medical charts noting that they had a partner, 41 (49%) had a partner in the United States; 30 (36%) did not have their partner in the United States, and 12 (14%) had unknown partner location status. Of the 41 who reported having a partner in the United States, 11 (27%) had it noted in their medical chart that their partner was also HIV-infected; 20 (49%) had partners who were reportedly uninfected; and 10 (24%) had reported no information on partner’s HIV status. Overall, 80 (51%) reported having children, and 30 (19%) had no children; 47 (30%) had no information regarding children. Among the 80 refugees with children, three (4%) had medical charts that noted having children with HIV infection; 37 (46%) had children without HIV infection; and 40 (50%) had no record of children’s HIV-status. Among 74 female refugees, two (3%) were pregnant on initial presentation; 20 (27%) became pregnant sometime after their initial presentation to a health clinic. After arrival, the median time to pregnancy for 16 females with both pregnancy and arrival dates documented was 2.2 years (range, 0.1–7.5 years).

**Table 1:** Demographic Characteristics of 184 Refugees Diagnosed with HIV Infection, and 157 HIV-Infected Refugees with Medical Chart Review for 2000–2007, Minnesota.

Characteristic	Total with HIV waiver or diagnosed	Total screened, chart not abstracted	Eligible Adults with HIV waiver or diagnosed in US *	Eligible Adults with Refugee Health assessment screening *	Adults enrolled, charts abstracted
	No. (%)	No. (%)	No. (%)	No. (%)	No. (%)
**Total**	184 (100)	25 (100)	174 (100)	165 (100)	157 (100)
**Male**	96 (52)	13 (52)	96 (55)	92 (56)	83 (53)
**Female**	88 (48)	12 (48)	78 (45)	73 (44)	74 (47)
**Age (years)**					
<5	1 (1)	1 (4)	0 (0)	0 (0)	0 (0)
5–14	3 (2)	3 (12)	0 (0)	0 (0)	0 (0)
15–24	79 (43)	14 (56)	75 (43)	70 (42)	65 (41)
25–44	61 (33)	4 (16)	61 (35)	58 (35)	56 (36)
45–64	36 (20)	2 (8)	34 (20)	33 (20)	33 (21)
≥65	4 (2)	1 (4)	4 (2)	4 (2)	3 (2)
**Region of origin**					
Sub-Saharan Africa	178 (97)	22 (88)	169 (97)	161 (98)	154 (98)
South, Southeast Asia	6 (3)	3 (12)	5 (3)	4 (2)	3 (2)
**Year of arrival**					
2000	27 (15)	6 (24)	24 (14)	24 (15)	21 (13)
2001	19 (10)	0 (0)	19 (11)	19 (12)	19 (12)
2002	0 (0)	0 (0)	0 (0)	0 (0)	0 (0)
2003	20 (11)	0 (0)	20 (11)	18 (11)	19 (12)
2004	48 (26)	5 (20)	46 (26)	45 (27)	43 (27)
2005	26 (14)	5 (20)	24 (14)	23 (14)	21 (13)
2006	33 (18)	8 (32)	30 (17)	26 (16)	24 (15)
2007	11 (6)	1 (4)	11 (6)	10 (6)	10 (6)

* Excludes two refugees who arrived w/ HIV waiver but tested negative in the US.

### 3.1. HIV/AIDS Treatment and Clinical Status

Among the 157 HIV-infected refugees who had medical charts available for review, 131 (83%) had sufficient clinical information to categorize the patient by WHO clinical stages as described previously. Among these, 108 (82%) had CD4^+^ count information available for ≤1 year after their initial clinical visit (Table 2). The WHO stages at first provider visit were Stage I (asymptomatic, 59%), Stage II (mild, 11%), Stage III (advanced, 10%), and Stage IV (severe, 3%); 26 of the 157 (17%) had unknown or unrecorded CD4^+^ counts on their medical charts. The median CD4^+^ count of those who were asymptomatic, mildly symptomatic, or had severe symptoms increased during the 1-year follow-up periods, although the range of CD4^+ ^counts varied widely. Before arrival, 6 (4%) reported having received or were receiving standard antiretroviral therapy (ART). After arrival, 109 (69%) received ART. 

**Table 2:** Estimated World Health Organization Clinical Stage of HIV/AIDS and Initial CD4^+^ Counts for HIV-Infected Refugees at First Provider Visit After and Subsequent CD4^+^ Counts.

		Initial CD4^+^ Count		1st Month CD4^+^ Count		6th Month CD4^+^ Count		1st Year CD4^+^ Count	
Initial clinical stage	N (%)	No.	Median (Range)	No.	Median (Range)	No.	Median (Range)	No.	Median (Range)
Asymptomatic	93 (59)	91	300 (32–1,084)	79	320 (80–1,032)	77	339 (50–1,146)	74	352 (65–1,291)
Mild symptoms	18 (11)	18	181 (15–440)	17	203 (47–375)	16	267 (52–517)	15	287 (129–547)
Advanced symptoms	16 (10)	16	271 (7–452)	16	233 (7–590)	15	251 (64–581)	15	382 (47–503)
Severe symptoms	4 (3)	3	10 (3–66)	4	80 (5–140)	4	114 (69–156)	4	138 (110–192)
Unknown	26 (17)	17	283 (76–1,921)	11	340 (97–665)	12	319 (153–636)	11	321 (215–770)
Total	157 (100)	145		127		124		119	

### 3.2. Infections and Opportunistic Infections

Twenty-one HIV-infected refugees (13%) received diagnoses with ≥1 opportunistic infection (OI), which included candidiasis (n = 9), pulmonary (n = 6) and disseminated TB (n = 2), *Mycobacterium avium* complex (n = 7), *Pneumocystis jiroveci* (n = 3), Kaposi’s sarcoma (n = 2), toxoplasmosis (n = 1), cryptosporidiosis (n = 1), and histoplasmosis (n = 1). Among the 21 refugees who experienced an OI, 1 did so before arrival; eight were reported to have an OI 1–3 months after their arrival; and 13 experienced an OI 4 months–7 years after their arrival in the United States. Three (2%) died, all from AIDS-related causes, between 3–4 years after arrival into the United States; none were reported to have received ART before arrival and two were reported to have received ART after arrival.

During 2000–2007, there were 161 HIV-infected refugees and 12,287 HIV-uninfected refugees who arrived in Minnesota from sub-Saharan African countries and received an initial refugee health assessment (Table 3). This included four refugees who tested positive for HIV overseas but were found to be HIV-uninfected upon re-testing in the US, and one refugee found to be HIV-infected in the US at health screening assessment. Coinfections for HIV-infected refugees as well as those same infections for HIV-uninfected refugees were common at initial health screening visits for refugees resettled from sub-Saharan Africa (Table 3). TB disease was more common among HIV-infected refugees, compared with HIV-uninfected refugees. The proportion of HIV-infected refugees with hepatitis B infection was similar to HIV-uninfected refugees. Parasitic infections were slightly less common among HIV-infected refugees, compared with HIV-uninfected refugees. Syphilis was more frequently observed among HIV-infected refugees, compared with HIV-uninfected refugees, and none of the HIV-infected refugees included were coinfected with chlamydia or gonorrhea.

**Table 3:** Demographics and Coinfections Identified during Routine Refugee Health Screening Assessment Comparing HIV-Infected to HIV-Uninfected Refugees from Sub-Saharan Africa Resettled to Minnesota during 2000–2007.

Characteristic	HIV-Infected Refugees (n = 161)		HIV-Uninfected Refugees (n = 12,287)		*P* Value *
	No.	(%)	No.	(%)	
**Sex**					0.300 *
Male	89	(55.3)	6,297	(51.2)	
Female	72	(44.7)	6,009	(48.8)	
**Age (y** **ears)**					<0.001 *
17–24	75	(46.6)	7,273	(59.1)	
25–44	51	(31.7)	2,091	(17.0)	
45–64	31	(19.3)	2,268	(18.4)	
≥65	4	(2.5)	674	(5.5)	
**Year of arrival**					0.004 *
2000	24	(14.9)	1,557	(12.7)	
2001	19	(11.8)	1,135	(9.2)	
2002	0	(0)	257	(2.1)	
2003	18	(11.2)	1,239	(10.1)	
2004	45	(28.0)	2,190	(17.8)	
2005	19	(11.8)	1,994	(16.2)	
2006	26	(16.2)	2,684	(21.8)	
2007	10	(6.2)	1,250	(10.2)	
**Time to arrival screening**					<0.001 *
≤29 days	140	(87.0)	5,024	(40.8)	
30–59 days	19	(11.8)	4,626	(37.6)	
60–90 days	1	(0.6)	1,951	(15.9)	
≥91 days	1	(0.6)	705	(5.7)	
**Tuberculosis**					<0.001
Disease	5	(3.1)	206	(1.7)	
Latent infection	55	(34.2)	6,978	(56.8)	
No tuberculosis	91	(56.5)	4,280	(34.8)	
Not tested	10	(6.2)	823	(6.7)	
**Hepatitis B**					0.809 *
Positive test hepatitis B	16	(9.9)	1,177	(9.6)	
Negative test hepatitis B	143	(88.8)	10,845	(88.3)	
Not tested	2	(1.2)	265	(2.2)	
**Parasitic infections**					0.041 *
Any parasitic infections §	12	(7.5)	1,501	(12.2)	
No parasitic infections	146	(90.7)	10,041	(81.6)	
Not tested	3	(1.9)	764	(6.2)	
Schistosomiasis	5	(3.1)	175	(1.4)	0.192 †
Giardiasis	4	(2.5)	368	(3.0)	0.822 †
Amebiasis	2	(1.2)	250	(2.0)	0.589 †
Strongyloidiasis	1	(0.6)	26	(0.2)	0.324 †
*Taenia* species infection	0	(0)	10	(<0.1)	>0.999 †
Other	0	(0)	954 **	(7.8)	<0.001 †
**Sexually transmitted infections**					
Syphilis	6	(3.7)	162 ††	(1.3)	0.018
No syphilis	117	(72.7)	9,600	(78.0)	
Not tested	38	(23.6)	2,544	(20.7)	>0.999
Gonorrhea	0	(0)	4 §§	(<0.1)	
No gonorrhea	18	(11.2)	1,080	(8.8)	
Not tested	143	(88.8)	11,222	(91.2)	>0.999
Chlamydia	0	(0)	3	(0.02 )	
No chlamydia	24	(14.9)	1,112	(9.0)	
Not tested	137	(85.1)	11,191	(90.9)	

* *Χ*^2^ test used; all others Fisher’s exact test. § A person might have been coinfected with ≥2 types of parasites. ** 505 *Blastocystis hominis,* 407 *Trichuris trichiura*, 119 *Dientamoeba*, 30 Hookworm species, 25 *Ascaris* lumbricoides, two *Helicobacter pylori*, one *Cryptosporidium*, 12 other. † Compared to no parasitic infection. †† one coinfected with gonorrhea/chlamydia/syphilis. §§ one coinfected with gonorrhea/chlamydia.

**Figure 2:**
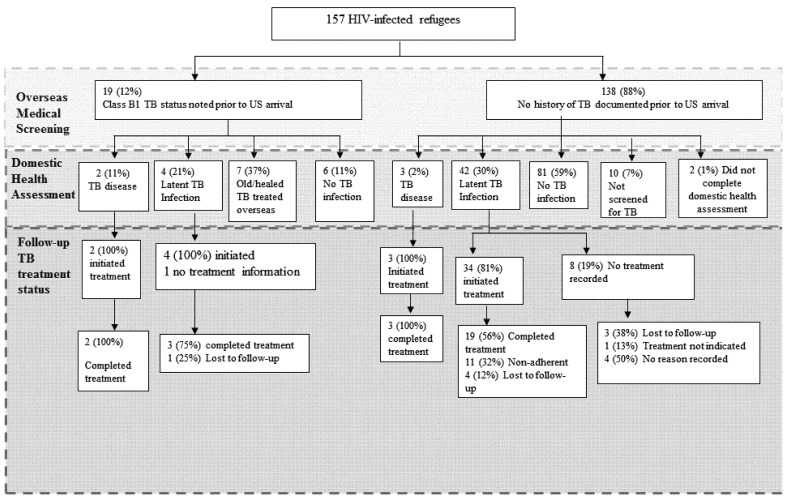
Tuberculosis (TB) Diagnosis and Treatment Status Among HIV-Infected Refugees Resettled to Minnesota, 2000–2007.

Among the 157 refugees with charts reviewed, 51 (32%) were diagnosed with TB disease or LTBI at their domestic health assessment with an additional eight (5%) refugees developing TB disease after arrival. Nineteen (12%) had a record of Class B1 TB status before US arrival (Figure 2). Two of 19 (11%) HIV-infected refugees with a history of TB also had TB disease diagnosed at domestic health assessment; both completed treatment. Four Class B1 arrivals (21%) were diagnosed with LTBI; all four initiated treatment and three (75%) completed treatment. Among HIV-infected refugees with no TB Class upon arrival three (2%) of 138 were diagnosed with active TB disease at their domestic health assessment; all three completed treatment. 

Among 42 HIV-infected refugees with no TB class who were diagnosed with LTBI at domestic health assessment, 34 (81%) initiated treatment and 19 (56%) of those completed treatment. Of those who did not complete treatment, eleven (26%) were non-adherent to prescribed treatment, four (10%) were lost to follow-up, and eight (19%) did not have a treatment start-date recorded. Reasons for lack of start-date were loss to follow-up (38%), treatment was determined to not be indicated by the provider (13%), or no reason was recorded (63%). 

## 4. Discussion and Conclusions

In Minnesota, during the period prior to the cessation of the waiver requirement, HIV-infected refugees arrived in Minnesota at varying stages from asymptomatic infection to advanced disease. A majority had no prior record of ART; coinfections detected at post-arrival domestic health screening were common; and three AIDS-related deaths occurred. Two HIV-infected refugee women were pregnant at arrival, and 20 became pregnant after arrival, thus requiring specialized care to prevent mother-to-child HIV transmission. These findings reinforce the importance of providing information regarding HIV-infection to refugee populations [8]. In addition, these data may serve as useful baseline comparison for the evaluation of changing practices and guidelines of HIV-testing. 

Before 2010, documentation of HIV infection through the waiver program was the primary means of identifying HIV-infected refugees prior to arrival. The impact of the 2009 rule change on refugee health, particularly those who are HIV-positive, is unknown. Because early identification and appropriate initiation of therapy for HIV is associated with decreased morbidity and improved overall survival [11], collaboration between refugee health programs and HIV counseling and testing services will be of increased importance. Current CDC guidelines recommend HIV screening in health-care settings for all persons aged 13–64 years, including refugees [3]. Voluntary screening is encouraged by CDC for all refugees on arrival, when counseling and referrals for care, treatment, and preventive services are available [6]. These changes are in accordance with the United Nations High Commissioner for Refugees (UNHCR), WHO, and United Nations Program on HIV/AIDS recommendations regarding HIV testing and counseling in health facilities for refugees, internally displaced persons, and other persons of concern to UNCHR [12]. 

Identifying health needs and providing care to resettled refugees fits within the framework of upholding health as a human right. Data regarding HIV prevalence and risks for acquiring HIV infection among refugee populations are sparse [13,14], and testing might not be available before arrival. After the change from required HIV testing, timely and accurate HIV diagnosis and treatment is of increased importance. Therefore, the arrival refugee medical examination provides an optimal opportunity to identify HIV infection among refugees and provide treatment. Coinfections are common and morbidity likely can be reduced with earlier diagnosis and treatment of HIV infection. As illustrated by the frequency of pregnancy among this population, the early identification of HIV after arrival can also reduce the likelihood of perinatal HIV transmission. Continued collaboration between public health agencies and health care providers will be necessary to follow and assess refugees’ health status, including HIV infection, in Minnesota and throughout the United States.

Similar to other reports investigating the health of HIV-infected refugees, coinfections were commonly detected [7,15,16]. The high prevalence of TB among refugees described in this analysis is expected, considering that TB is a common coinfection among HIV-infected persons and in sub-Saharan Africa [17,18]. Knowing a patient’s HIV status may influence clinicians suspicion and approach to TB infection and disease diagnosis. Moving forward, with the rule change, clinicians will not have the advantage of knowing the HIV status prior to arrival, making post-arrival screening important in detecting both HIV and TB disease.

During the period covered by chart reviews, US immigrant and refugee TB screening requirements were updated from 1991 requirements. In 1991, the requirements included a chest radiograph and three sputum samples (acid-fast–bacilli stain only) for disease detection. In 2007, these technical instructions were updated to require screening to include mycobacterial culture and completion of directly observed therapy before US arrival for anyone with TB disease [19]. These data indicate that a substantial proportion (52%) of those with LTBI detected during their domestic health assessment did not complete treatment or treatment outcome was missing; LTBI patients do not have access to directly observed therapy making it challenging for public health and/or health care providers to monitor their treatment through completion. Linguistic or socio-cultural barriers and loss to follow-up due to out-migration are additional challenges that can disrupt LTBI treatment adherence. LTBI and treatment completion are not reportable to the TB surveillance or refugee health programs, which may have led to underreporting of treatment outcomes. In recent years, the refugee health program at the Minnesota Department of Health has improved its processes to actively track LTBI treatment outcomes. For refugees arriving from 2008 to 2010, the program has seen an increase in the proportion of refugees who follow LTBI treatment through to completion, due in part to improved tracking and reporting; 78% of all refugees who completed LTBI treatment, compared to only 48% during 2000–2007. However, this is still inadequate, particularly for those with HIV infection where the TB reactivation rate is excessive.

These data, based on medical chart review of resettled refugees, provide a unique summary of the health status of HIV-infected refugees during a period when HIV-infection status was known before US arrival. However, this study has certain limitations. As a retrospective medical chart review, certain clinical details might not be recorded in medical charts; others might have been missed during the process of chart abstraction; and patients might have sought health care elsewhere from the site of medical chart abstraction. To maintain a simplified data collection tool, certain details (e.g., the date that ART was prescribed) and treatment rationale were not ascertained. This review did not include psychosocial components, as others have included [16]. Despite these limitations, our findings provide additional information given the limited data that exist regarding HIV-infection among refugee populations and can serve as useful baseline comparisons in lieu of changing practices and guidelines of HIV-testing and availability of ART. To complement medical chart abstractions, future evaluations of health status of HIV-infected refugee populations should include culturally appropriate patient interviews to better understand their knowledge, attitudes, and beliefs regarding HIV/AIDS testing and care.

In Minnesota, evaluating the health of all refugees through a health screening assessment, regardless of HIV-infection status is a routine MDH activity, provides a means for determining care and prevention needs, and includes constant interaction within MDH programs (e.g., TB prevention services follow-up). During 2000–2007, the refugee health screening rate was 94.8% of 29,182 eligible resettled refugees. Refugees are ineligible if they move out of state or to an unknown location or have incorrect contact information. In lieu of the waiver program, routine HIV screening is the most efficient way to detect HIV infection and provide appropriate and timely care.
